# The Emerging Role of Zinc in the Pathogenesis of Multiple Sclerosis

**DOI:** 10.3390/ijms18102070

**Published:** 2017-09-28

**Authors:** Bo Young Choi, Jong Won Jung, Sang Won Suh

**Affiliations:** Department of Physiology, Hallym University, College of Medicine, Chuncheon 24252, Korea; bychoi@hallym.ac.kr (B.Y.C.); jwj0825@gmail.com (J.W.J.)

**Keywords:** multiple sclerosis, zinc, EAE, MMP-9 activation, BBB disruption, microglia activation

## Abstract

Our lab has previously demonstrated that multiple sclerosis-induced spinal cord white matter damage and motor deficits are mediated by the pathological disruption of zinc homeostasis. Abnormal vesicular zinc release and intracellular zinc accumulation may mediate several steps in the pathophysiological processes of multiple sclerosis (MS), such as matrix metallopeptidase 9 (MMP-9) activation, blood-brain barrier (BBB) disruption, and subsequent immune cell infiltration from peripheral systems. Oral administration of a zinc chelator decreased BBB disruption, immune cell infiltration, and spinal white matter myelin destruction. Therefore, we hypothesized that zinc released into the extracellular space during MS progression is involved in destruction of the myelin sheath in spinal cord white mater and in generation of motor deficits. To confirm our previous study, we employed zinc transporter 3 (*ZnT3*) knockout mice to test whether vesicular zinc depletion shows protective effects on multiple sclerosis-induced white matter damage and motor deficits. *ZnT3* gene deletion profoundly reduced the daily clinical score of experimental autoimmune encephalomyelitis (EAE) by suppression of inflammation and demyelination in the spinal cord. *ZnT3* gene deletion also remarkably inhibited formation of multiple sclerosis-associated aberrant synaptic zinc patches, MMP-9 activation, and BBB disruption. These two studies strongly support our hypothesis that zinc release from presynaptic terminals may be involved in multiple sclerosis pathogenesis. Further studies will no doubt continue to add mechanistic detail to this process and with luck, clarify how these observations may lead to development of novel therapeutic approaches for the treatment of multiple sclerosis.

## 1. Introduction

Multiple sclerosis (MS) is characterized by numerous maladaptive or abnormal responses of the immune system, which result in the erroneous and progressive destruction of central nervous system (CNS) structures, including those of the brain and spinal cord. MS is an autoimmune disease characterized by damage to the myelin sheath that entwines and insulates the naked nerve fibers, allowing efficient conduction of neural impulses. This demyelinating process is initiated when T cells become sensitized to endogenous myelin proteins and begin to erroneously attack myelinated structures in the central and peripheral nervous system. Since myelin damage slows the conduction velocity of action potentials along the length of the nerve, communication between the brain and other parts of body is impaired. Consequently, most MS patients experience muscle weakness and difficulties with coordination, resulting in irreversible neurologic impairment [1]. Effective treatment for MS has not been developed yet, while anti-inflammatory therapies have been shown to delay MS progression [2]. However, a majority of available MS treatments have shown side effects, and there is little confirmation of the efficacy of personal therapeutic agents. Therefore, defining a full mechanism of MS will enable the design of respective treatment plans against disease heterogeneity.

Although it is currently believed that the interaction of several different elements (such as immunologic, environmental, infectious and genetic factors) may be responsible for the disease [3], the exact cause of MS remains unknown. In other words, pathogenesis of MS might be the result of an interaction between several factors [1]. For instance, Baranzini, et al. reported discordance of MS in their research on monozygotic twins, suggesting that genetic factors cannot fully explain MS incidence [4].

Several studies investigating risk factors of MS pathogenesis have been published. Viral infection has been suggested as playing significant role in MS development by modulating the immune system, resulting in susceptibility to MS [5]. Several lines of evidence reported that MS patients have immunoglobulin G (IgG) antibodies against viruses such as the Epstein-Barr virus (EBV), and varicella zoster virus (VZV) [6,7]. Human herpesvirus 6 (HHV6) is also found to have significantly positive relationship with MS [8], while cytomegalovirus (CMV) infection seems to have negative association [5]. On the other hand, some researchers suggest that parasite infection provides protection against MS [9,10]. MS patients in Turkey who were seropositive to Toxoplasma gondii showed better clinical outcomes as compared to seronegative MS patients [10]. Also, excretory products of *Trichruis suis* have been shown to reduce disease severity in experimental autoimmune encephalomyelitis (EAE), a well-known animal model for MS [10]. These findings hypothesize that immunomodulation by parasite infection is protective with respect to MS, but the precise mechanism in terms of how these organisms modulate the immune system is elusive. Moreover, noninfectious environmental factors such as smoking and low vitamin D level have been suggested to be MS risk factors, while statistical evaluation concluded that biomarkers associated with EBV infection, infectious mononucleosis, and smoking are all risk factors with epidemiological significance [11].

According to the last proposal on MS subtype classification by the International Advisory Committee on Clinical Trials of MS, MS is classified into 4 subtypes by clinical patterns presented by patients: clinically isolated syndrome (CIS), relapsing-remitting MS (RRMS), secondary progressive MS (SPMS), and primary progressive MS (PPMS). CIS stands for a subtype that involves only a single episode of the disease, without a secondary attack. RRMS is the most common subtype of MS, and is characterized by relapsing MS symptoms followed by a period with no disease progression. SPMS is defined as a subtype in which a period of RRMS precedes a stage of disease progression. PPMS is a progressive subtype without relapse symptoms [12]. The clinical significance of this classification comes from the report that each subtype responds differently to available treatments [13,14], and it should be noted that each subtype can transform to another subtype as disease life is prolonged.

Experimental autoimmune encephalomyelitis (EAE), used in exploration of neuroinflammatory pathology, is a common disease model of MS. However, the key difference between EAE and MS is that EAE has a known etiology, while MS does not. [15]. EAE can be induced in monkeys and rodents by immunization with tissue or molecules that originate from the CNS, such as myelin oligodendrocyte glycoprotein (MOG), myelin basic protein (MBP) and proteolipid protein (PLP). It may also be induced by transferring MOG-, MBP-, or PLP-specific T lymphocytes as an alternative to direct induction [16,17]. Depending on the antigen used and the genetic make-up of the animal, rodents can display a monophasic bout of EAE, a relapsing-remitting form, or chronic EAE. The typical susceptible rodent will debut with clinical symptoms in a prodromal period of 10–15 days after immunization, followed by ascending paralysis beginning in the tail and hind limbs with progress to the forelimbs, concurrent with weight loss.

## 2. Zinc in the Central Nervous System

Zinc is one of the most abundant trace elements in the human brain and serves diverse functions in our body. Zinc acts as a cofactor of numerous enzymes like matrix metalloproteinases (MMPs) and constitutes several proteins, including MBP. Its distribution is focused in certain brain regions such as the cerebral cortex, the hippocampus and the amygdala [18], indicating that zinc may participate in processing cognition, memory and emotion [19]. Also, zinc has been reported to play certain roles in both the innate and adaptive immune system [20].

Reflecting its diverse functions, zinc transporters (ZnTs), channels and intracellular binding proteins organize a network that participates in translocation and stabilization of zinc in the CNS [21,22,23]. ZnT resembles Zn^2+^/H^+^ or K^+^ antiporters in terms of function, and lowers cytosolic Zn^2+^ levels [24], while Zn^2+^-importing proteins (ZIP) increase cytosolic Zn^2+^ levels by translocating Zn^2+^ from the extracellular space or organelles [25]. Also, metallothionein (MT) is a family of intracellular proteins that binds zinc and acts as a zinc buffer [26,27]. The MT family consists of four isoforms. MT-1 and MT-2 are ubiquitously expressed forms [28] and MT-3 is exclusively expressed in neurons and testes [29,30], while squamous epithelial cells express MT-4 as they are differentiating [31]. The significance of MT in neuronal pathology has been previously demonstrated. A considerable amount of Zn^2+^ is released from MTs on exposure to oxidative stress, mediating neuronal death in seizures or ischemia. In addition, Penkowa and Hidalgo reported that MT-2 can be a potential target of treatment for MS, as it was shown to reduce cytokine expression in the CNS and apoptotic neuronal death in an EAE model [32].

Chelatable zinc locates to the synaptic vesicle, and *ZnT3* is suggested to play a key role in this physiologic state [33], as vesicular zinc was not detected in *ZnT3* knockout (KO) mice [34]. The *ZnT3* gene (*Slc30a3*), a critical zinc transporter, was originally characterized by Palmiter, et al. [35]. *ZnT3* is localized to glutamatergic synapses in the brain, including the entorhinal cortex, the amygdala and the hippocampus. In addition, it is selectively located in the vesicles of zinc-secreting neurons, and is responsible for most of the zinc released into the synapse [36,37]. *ZnT3* has been shown to be essential for transport of synaptic vesicular Zn^2+^ [34,38] and therefore may influence the downstream effects of synaptic Zn^2+^ on several signaling pathways. For instance, stimulation of zinc-containing neurons leads to synaptic release of vesicular zinc [39,40]. On release from the presynaptic terminal, vesicular zinc enters postsynaptic neurons via such channels as the α-amino-3-hydroxyl-5-methyl-4-isoxazole-propionate (AMPA)/kainate channel, the *N*-methyl-d-aspartate (NMDA) channel, and the voltage-sensitive Ca^2+^ channel (VSCC) [41].

## 3. The Molecular Mechanisms of Zinc-Induced Neurotoxicity

Vesicular zinc release into synaptic cleft is suggested to induce neuron death in neurologically stressful conditions [42,43], such as hypoglycemia [44,45], seizure [46], cerebral ischemia [47], and traumatic brain injury [48]. Zinc-induced neuronal death in hypoglycemia proceeds via the following steps. First, glucose reperfusion after hypoglycemia initiates production of nitric oxide, which releases vesicular zinc from presynaptic neurons [49]. Zinc then translocate into postsynaptic neurons through glutamate receptors [42,50] and voltage-sensitive calcium channels [41]. As a high intracellular zinc concentration is achieved in neurons, zinc has been reported to induce reactive oxygen species (ROS) production by mitochondria [51] and by NADPH oxidase activation [52]. ROS have been shown to mediate neuronal death by inducing oxidative stress in DNA, releasing zinc bound to MT and further promoting intracellular zinc increase, and activating poly(ADP-ribose) polymerase-1 (PARP-1). PARP-1 is an enzyme located in the nucleus, and its overactivation changes mitochondrial permeability and the release of apoptosis-inducing factor, eventually inducing neurotoxicity [53]. In particular, zinc chelation after hypoglycemia and glucose reperfusion reduced ROS production, PARP-1 activation, and neuronal death. *ZnT3* gene deletion, which depletes zinc in synaptic vesicles, also reduced ROS production and neuronal death [49]. These findings suggest vesicular zinc is a key mediator of neuronal death induced by acute brain injury.

A relationship between intracellular zinc release and neuronal death has been suggested by other research groups [54]. Aizenman, et al. reported that zinc release from MT can be initiated by exposure to oxidative stress, and it leads to neuronal death, as a cell-permeant zinc chelator relieves neurotoxicity of 2,2′-dithiodipyridine (DTDP), a membrane-permeant oxidizing agent. This report hypothesizes the possible role of intracellular zinc release in mediating neuronal injury [55]. The underlying mechanism of neuronal death by endogenous zinc was elaborated by groups of researchers, as intracellular zinc release was shown to activate the mitogen-activated protein kinase (MAPK) pathway, which leads to neuronal apoptosis [56,57]. Zinc release from intracellular compartments drives its significance as a mediator of neuronal injury, as zinc accumulation was detected in degenerating neurons in a *ZnT3* KO seizure model, implicating sources of zinc other than synaptic vesicles [58]. However, a relationship between intracellular zinc release and MS is yet to be proven. Intracellular zinc release may participate in EAE pathogenesis, perhaps resembling synaptic zinc release in its mechanism, as both events lead to increased intracellular zinc levels.

## 4. The Possible Role of Zinc in MS

### 4.1. Vesicular Zinc as a Key Mediator of Neuronal Death in MS

**Figure 1:**
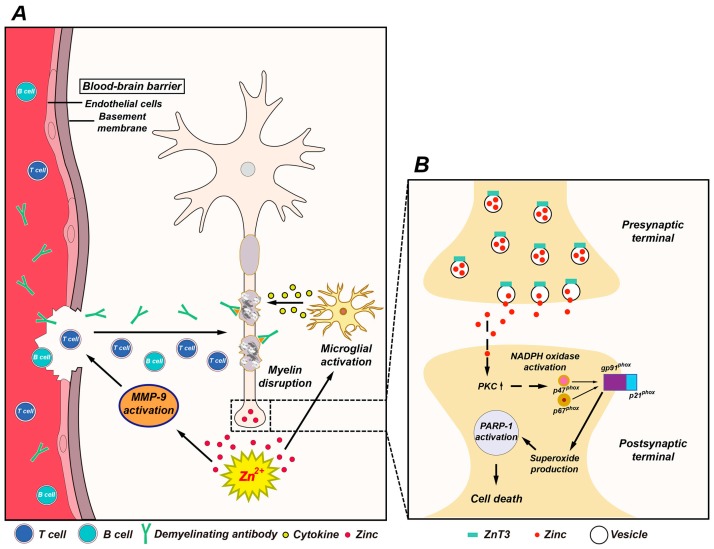
Possible association of zinc with multiple sclerosis (MS) pathogenesis. (**A**) This schematic drawing indicates several ways in which zinc may influence MS pathogenesis. Released zinc may induce matrix metallopeptidase-9 (MMP-9)-dependent blood-brain barrier (BBB) disruption. This may further enhance the migration of autoreactive immune cells and demyelinating antibodies by degrading extracellular-matrix macromolecules. In addition, released zinc may activate microglia and then proinflammatory cytokines released by activated microglia cause damage to the myelin sheath. (**B**) Mechanism of zinc-induced neuronal damage in MS. Cytoplasmic influx of synaptically released zinc stimulates protein kinase C (PKC), NADPH oxidases, and superoxide-generating enzyme. Superoxide production activates poly(ADP-ribose) polymerase-1 (PARP-1) in the nucleus, leading to cell death.

Previously, our lab demonstrated that EAE-induced spinal cord white matter damage and motor deficits were mediated by pathological disruption of zinc homeostasis. Abnormal vesicular zinc release and intracellular zinc accumulation may mediate several steps of the EAE pathophysiological process, such as matrix metallopeptidase-9 (MMP-9) activation, blood-brain barrier (BBB) disruption, and subsequent immune cell infiltration from peripheral systems. Thus, oral administration of a zinc chelator decreased BBB disruption, immune cell infiltration, and spinal white matter myelin destruction. Therefore, we hypothesized that zinc released into the extracellular space during MS progression is involved in destruction of the myelin sheath in the spinal cord white matter and in generation of motor deficits [59,60] (Figure 1).

However, our previous study did not fully address how extracellular zinc causes this sequence of events to be involved in myelin damage. Several lines of evidence have demonstrated that zinc alone induces NADPH oxidase activation in neurons and results in reactive oxygen species production. Our lab demonstrated that translocation of the p47phox and p67phox subunits from the cytoplasm to the plasma membrane occurs upon glucose deprivation/glucose reperfusion, and that this translocation is prevented by zinc chelation [45]. Since reporting this observation, zinc-induced neuronal NADPH oxidase activation in neuronal cultures has been demonstrated by various other groups [52,61]. Thus, these results suggest that zinc release may be an intermediary event in NADPH oxidase activation-induced ROS production in several types of CNS injury.

**Figure 2:**
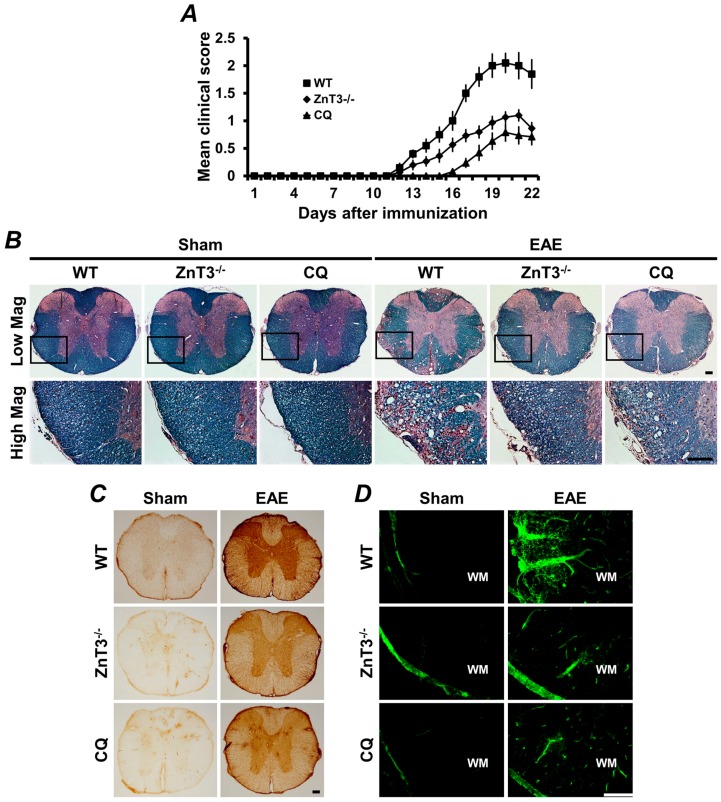
Experimental autoimmune encephalomyelitis (EAE)-induced clinical and neuropathological changes were reduced by *ZnT3* gene deletion or zinc chelation. (**A**) The clinical score of EAE was significantly reduced in *ZnT3* knockout and clioquinol (CQ)-treated mice. Data are with respect to mean SEM (*n* = 10–21) * *p* < 0.05 compared with wild type (WT) immunized mice. (**B**) Representative images showed luxol fast blue (LFB) staining in white matter of the thoracic spinal cord 21 days after the initial MOG_35–55_ immunization. LFB staining of the spinal cord represents extensive demyelination in the WT immunized mice. However, EAE-induced demyelination was remarkably reduced in the *ZnT3* knockout and CQ-treated mice. Scale bar = 100 μm. (**C**) Representative images showed immunoglobulin G (IgG) extravasation in the thoracic spinal cord of EAE mice. Sham-operated mice revealed only weak IgG immunoreactivity (brown color) primarily confined to the BBB-deficient area. WT immunized mice revealed prominent extravasation of IgG throughout the parenchyma of the spinal cord. However, *ZnT3* knockout and CQ-treated mice showed significant reduction of IgG immunoreactivity. Scale bar = 100 μm. (**D**) Fluorescence photomicrographs represented MMP-9 activity in the white matter of sham-operated or EAE mice. MMP-9 was localized by in situ zymography. A weak fluorescence signal was detected in sham-operated mice. However, the fluorescence intensity significantly increased in the white matter of the spinal cord in EAE-induced WT mice. This increased fluorescence intensity was remarkably reduced in *ZnT3* knockout and CQ-treated mice. Scale bar = 100 μm. MOG: myelin oligodendrocyte glycoprotein.

To confirm our previous study, we employed oral treatment with clioquinol (CQ), a well-characterized zinc chelator, and *ZnT3* KO mice to test whether vesicular zinc depletion shows protective effects on EAE-induced white matter damage and motor deficits. *ZnT3* gene deletion or CQ treatment profoundly reduced the daily clinical score of EAE. The *ZnT3* gene deletion or CQ-mediated inhibition of the clinical course of EAE was accompanied by suppression of inflammation and demyelination in the spinal cord. The motor deficit-accompanying neuropathological changes associated with EAE were less severe in *ZnT3* KO or CQ-treated mice. This reduction in motor deficit was accompanied by coincident reduction in demyelination and infiltration of encephalitogenic immune cells, including cluster of differentiation (CD4+ T cells, CD8+ T cells, CD20+ B cells) and F4/80+ microglia in the spinal cord. These results demonstrate that *ZnT3* gene deletion inhibits the clinical features and neuropathological changes associated with EAE. *ZnT3* gene deletion or CQ treatment also remarkably inhibited formation of EAE-associated aberrant synaptic zinc patches, MMP-9 activation, and BBB disruption. Therefore, amelioration of EAE-induced clinical and neuropathological changes by *ZnT3* gene deletion or zinc chelation suggests that vesicular zinc may be involved in several steps of MS pathogenesis [59,62] (Figure 2). Overall, these results strongly support our hypothesis that zinc release from presynaptic terminals may be involved in EAE pathogenesis.

### 4.2. Role of Zinc in Oligodendrocyte Death

Intracellular zinc release may play a role in primary oligodendrocyte death, leading to demyelination, the main pathologic characteristic of MS. Oligodendrocytes are a type of glia that form myelin sheaths in the CNS, and have been found to be injured in MS lesions [1]. Oligodendrocytes are susceptible, and are thus vulnerable to oxidative stress [63]. AMPA/kainate and NMDA receptors are present on the oligodendrocyte membrane. In particular, AMPA receptors without a GluR2 subunit contribute to oligodendrocyte susceptibility to glutamate excitotoxicity, owing to their vulnerability to Ca^2+^ influx [64]. Several lines of evidence suggest intracellular zinc release as a possible event that leads to oligodendroglia death in MS. Mato, et al. reported that intracellular zinc release is a key mediator of glutamate-induced excitotoxic injury to oligodendrocytes [65]. Ca^2+^ accumulation in postsynaptic neuron due to AMPA receptor activation was shown to induce intracellular zinc release, in accordance with reports on neurons [66]. Furthermore, two intracellular sources from which Zn^2+^ is released were defined as being the same as in neurons: mitochondria and MT. Zn^2+^ is thus reported to induce mitochondrial depolarization and ROS production, then followed by downstream biochemical cascades such as the MAPK pathway and eventually, the PARP-1 apoptotic pathway [67]. As a result, zinc is suggested to contribute to AMPA-mediated oligodendrocyte death by inducing oxidative stress and PARP-1-mediated cell death [65]. In addition, several lines of evidence suggested peroxynitrite toxicity, mediated by intracellular zinc release, as another candidate mediating oligodendroglia death in MS [57,68,69]. Peroxynitrite is produced by the reaction of nitric oxide (NO) and superoxide. The clinical significance of nitric oxide and peroxynitrite on MS is suggested as an increased expression of inducible nitric-oxide synthase (iNOS), and this has been shown in MS lesions [70]. MS patients were reported to have increased levels of markers indicative of peroxynitrite formation: iNOS and nitrotyrosine [71]. It has been reported that peroxynitrite releases zinc from intracellular stores such as MT and mitochondria, and zinc sequentially activates extracellular signal-regulated kinase 1/2 (ERK1/2), 12-lipoxygenase (12-LOX), and ROS production, leading to oligodendroglia death [68]. Peroxynitrite-induced toxicity in oligodendrocytes was relieved when the zinc chelator TPEN (*N*,*N*,*N*′,*N*′-tetrakis(2-pyridylmethyl)ethylenediamine) was treated, indicating the role of zinc as a significant factor in its mechanism [68]. Peroxynitrite toxicity has also been reported to be mediated by protein kinase C (PKC) [72]. As PKC is reported to mediate zinc-induced neurotoxicity [73], it may also elucidate the pathway of zinc-induced oligodendroglia death.

## 5. Conclusions and Future Directions

The idea of zinc playing a significant role in MS pathogenesis may arise from its high concentration in the CNS and involvement in brain physiology. Such circumstances as acute brain injury and neurodegenerative diseases accompany zinc homeostasis disruption, elucidating the possibility of zinc as a target of their treatment. In particular, groups of researchers have shown that oxidative stress is the main mediator of zinc neuro- and glio-toxicity [74]. As oxidative stress is suggested as a main cause of MS [13,71], a possible role of zinc in MS pathology can be evaluated.

Role of zinc in oligodendroglia death has also been suggested by groups of researchers, and it seems to involve similar mechanisms induced by intracellular zinc release in neuronal death [57,65,68,69]. Thus, zinc may play an extensive role in MS pathogenesis by provoking neuronal and oligodendrocyte death.

However, the molecular mechanisms underlying those findings have not been fully defined. Thanks to pioneering studies however, we have obtained a glimpse of the complex networks. Research defining the mechanisms of intracellular zinc release and the subsequent contribution to myelin sheath injury in an EAE model is also needed. Moreover, elucidating the relationship between zinc and early stage-MS brain lesions will provide us with a deeper understanding of role of zinc in MS pathogenesis. Further studies will undoubtedly continue to add mechanistic detail to this process and with luck, clarify how these observations may lead to the development of novel therapeutic approaches for the treatment of MS.
